# Progesterone, cervical cerclage or cervical pessary to prevent preterm birth: a decision-making analysis of international guidelines

**DOI:** 10.1186/s12884-022-04584-4

**Published:** 2022-04-23

**Authors:** Putora Katharina, Hornung René, Kinkel Janis, Fischer Tina, Putora Paul Martin

**Affiliations:** 1 Department of Gynecology and Obstetrics, Kantonsspital St. Gallen, St. Gallen, Switzerland; 2 Department of Radiation Oncology, Kantonsspital St. Gallen, St. Gallen, Switzerland; 3 Department of Radiation Oncology, University of Bern, Bern, Switzerland

**Keywords:** Cerclage, Pessary, Progesterone, Preterm birth, Guidelines

## Abstract

**Objective:**

The aim of this study was to investigate guidelines on preterm birth, analyze decision-criteria, and to identify consensus and discrepancies among these guidelines.

**Design:**

Objective consensus analysis of guidelines.

**Sample:**

Ten international guidelines on preterm birth.

**Methods:**

Relevant decision criteria were singleton vs. twin pregnancy, history, cervical length, and cervical surgery / trauma or Mullerian anomaly. Eight treatment recommendations were extracted. For each decision-making criteria the most commonly recommended treatment was identified, and the level of consensus was evaluated.

**Main outcome measures:**

Consensus and Discrepancies among recommendations.

**Results:**

In a case of singleton pregnancies with no history of preterm birth and shortened cervix, most guidelines recommend progesterone. In singleton pregnancies with a positive history and shortened cervix, all guidelines recommend a cerclage as an option, alternative or conjunct to progesterone. The majority of the guidelines advise against treatment in twin pregnancies.

**Conclusions:**

A shortened cervix and a history of preterm birth are relevant in singleton pregnancies. In twins, most guidelines recommend no active treatment.

**Tweetable abstract:**

Among international guidelines a shortened cervix and a history of preterm birth are relevant in singleton pregnancies. With no history of preterm birth and with a shortened cervix most guidelines recommend progesterone treatment.

## Introduction

In clinical practice, physicians and patients often have multiple options to choose from - obstetrics is no exception [1]. The decision criteria may be based on scientific evidence or may be associated with the decision-maker’s attitude (physicians expertise, patient’s preference) or the setting, where the decision is made in [2].

Preterm birth is still the leading cause of perinatal death and disability [3]. Despite efforts to reduce prematurity in the last decades, prematurity is rising worldwide, currently ranging from 5 to 18% of live births [4, 5]. While experts continue to debate the optimal management, especially before viability, various strategies exist. Conservative approaches include screening for periodontal disease, reduction of physical activity or bed rest, antibiotic treatment of bacterial vaginosis or asymptomatic bacteriuria, and smoking cessation [6].

Invasive measures include the use of a cervical cerclage (cervical stitch) or a cervical pessary. A cerclage may be inserted as an emergency treatment, when the cervix dilates without contractions (physical examination indicated cerclage). A prophylactic cerclage may be indicated based on the patient’s history - after preterm delivery or preterm rupture of membranes before 34 weeks of gestation and before cervical dilatation occurs (referred to as history-indicated cerclage). An ultrasound-indicated cerclage is applied, when the shortening of the cervix gets evident during a planned ultrasound session e.g. during routine second trimester screening. Rescue cerclage aims to reclose the cervical os and prevent the exposure of the amnion to vaginal bacteria, while history and ultrasound indicated cerclages aim to mechanically support the cervical os, maintaining a biochemical barrier and inducing an inflammatory response. This procedure needs general or regional anesthesia and may cause complications such as iatrogenic rupture of membranes, preterm labor, or intra-amniotic infection, especially in the setting of physical examination indicated cerclage [7].

A cervical pessary can support cervical closure by deviating the uterocervical angle, resulting in relief of pressure on the internal os of the cervix [8]. The pessary is inserted in lithotomy position without anesthesia. In correct position, a cervical pessary is associated with more vaginal discharge, but typically does not induce discomfort to the patient. It is removed easily.

Another potential therapy includes vaginal or intramuscular application of progesterone. In most studies, dosages between 100 and 200 mg/d were applied vaginally.

Guidelines support clinicians in decision-making. For the management of pre-term birth, a multitude of guidelines is available. The aim of this study was to investigate current international guidelines on the prevention of pre-term birth before or around viability, analyze decision-making criteria relevant for treatment selection, and to identify consensus and discrepancies among these guidelines.

## Methods

The selection of international guidelines on the management of pre-term birth is based on a systematic review published by Medley et al. in 2018 [9] complimented by a search for more recent updates. Institutional guidelines were excluded from the analysis. Seven guidelines were eligible for our analysis and are either regional, national, or international guidelines. If societies had published more than one guideline on the topic, the most recent guideline was included into the analysis. An additional unstructured literature search was performed focusing on guidelines published after the publication by Medley et al. resulting in the addition of the guidelines by the *Arbeitsgemeinschaft der Wissenschaftlichen Medizinischen Fachgesellschaften* (Guideline of the DGGG, OEGGG and SGGG) (AWMF, [10, 11]), by the European Association of Perinatal Medicine (EAPM, [12]) and the FIGO (International Federation of Gynaecology and Obstetrics) working group [13–15]. The publication dates ranged from 2014 to 2021. Three were international, six were national and one was regional. One was global (FIGO), five were from Europe, two from Northern America, one from Japan and one from Queensland, Australia. The list of guidelines analyzed is presented in Table 1.

**Table 1:** List of evaluated guidelines

Abbreviation	Publisher / Title of guideline	Year	Ref.
**ACOG**	American College of Obstetricians and Gynecologists	2021 2014	Committee on Practice Bulletins—Obstetrics, The American College of Obstetricians and Gynecologists. Practice bulletin no. 234: prediction and prevention of spontaneous preterm birth [16] American College of Obstetricians and Gynecologists. ACOG Practice Bulletin No.142: Cerclage for the management of cervical insufficiency [17]
**AWMF**	Arbeitsgemeinschaft der Wissenschaftlichen Medizinischen Fachgesellschaften - Guideline of the DGGG, OEGGG and SGGG	2019	Prevention and Therapy of Preterm Birth. Part 1 with Recommendations on the Epidemiology, Etiology, Prediction, Primary and Secondary Prevention of Preterm Birth [10] Part 2 with Recommendations on the Tertiary Prevention of Preterm Birth and the Management of Preterm Premature Rupture of Membranes [11]
**CNGOF**	Collège National des Gynécologues et Obstétriciens Français	2016	Prévention de la prématurité spontanée et de ses conséquences (hors rupture des membranes) [18]
**EAPM**	European Association of Perinatal Medicine	2017	Preterm Labor and Birth [12]
**FIGO**	International Federation of Gynaecology and Obstetrics	2021	FIGO good practice recommendations on cervical cerclage for prevention of preterm birth [14] FIGO good practice recommendations on progestogens for prevention of preterm delivery [15] FIGO good practice recommendations on the use of pessary for reducing the frequency and improving outcomes of preterm birth [13]
**JOG**	Japan Society of Obstetrics and Gynecology and Japan Association of Obstetricians and Gynecologists	2014	Guidelines for obstetrical practice in Japan: Japan Society of Obstetrics and Gynecology (JSOG) and Japan Association of Obstetricians and Gynecologists (JAOG) 2014 edition [19]
**KCE**	Belgian Health Care Knowledge Centre	2014	Prevention of preterm birth in women at risk: selected topics [20]
**NICE**	National Institute for Health and Care Excellence	2015 2017 2019	Preterm labour and birth NG25 [21] Surveillance report (exceptional review) 2017- Preterm labour and birth (2015) NG25 [22] Twin and triplet pregnancy NG 137 [23]
**SOGC**	Society of Obstetricians and Gynaecologists of Canada	2019	No. 373-Cervical Insufficiency and Cervical Cerclage [24]
**Queensland**	Queensland Clinical Guidelines - Maternity and Neonatal Clinical Guideline	2020	Preterm labour and birth [25]

Recommendations from each guideline were extracted by two independent specialists in obstetrics and gynecology and were converted into decision trees. Decision trees are visualizations of complex decision pathways to facilitate the decision process and have been applied for the analysis of other guideline analyses before [26–29].

Standardized decision-making criteria were defined to allow comparison of the selected guidelines. The decision trees were transformed to implement these standardized decision criteria. The four criteria relevant for decision-making within these guidelines were type of pregnancy (singleton vs. twin pregnancy), history of preterm birth, cervical length, and history of cervical surgery / trauma or anatomical anomalies (S/T/A). Patients with a history of preterm delivery were assigned to one of the groups H0 through H3. Patients with no history of preterm birth, preterm premature rupture of membranes (PPROM) or late miscarriage were assigned to H0, those with a history of one or two previous preterm births, PPROMs or late miscarriages to H1, while patients with a history of three or more preterm births/PPROMs or late miscarriages were assigned to H2. Patients with a history of trachelectomy or a history of a previously failed cervical cerclage resulting in a preterm birth/late miscarriage were assigned to the H3 cohort. Cervical length (CL) was measured by ultrasound and the length was stated in mm. Cervical opening of more than 1 cm is represented as CL = 0.0 (cervical opening 1-4 cm). History of S/T/A was classified as yes or no.

Treatment options were extracted from each of the ten guidelines and assigned to the according combination of decision-making parameters. Total cervical occlusion (TCO) was only mentioned by a single guideline (AWMF) and was not further evaluated in the present analysis [30].

Five individual treatment recommendations were extracted from the analyzed guidelines. The acronym noTx represents no recommendation for treatment, C represents cervical cerclage, P – progesterone therapy (commonly applied through the vaginal and rarely through the parenteral route), Pess stands for cervical pessary, and aC for abdominal cerclage, respectively. In many situations, multiple options or combinations were recommended resulting in eight specific treatment options (including single treatments and various combinations of them). CorP leaves the choice of either cerclage or progesterone to the physician/patient, C&orP stands for either one of the two options or even the combination of both, P+/−Pess indicates the use of progesterone with or without the additional use of a cervical pessary. The absence of a specific recommendation was represented as noR – no recommendation. The various routes of applications as well as the various dosages of progesterone therapy were summarized under the term P. This included the vaginal application (micronized progesterone most commonly dosed as 100-200 mg/d as capsules, or 90 mg/d as vaginal gel) or intramuscular injection of progesterone (17-hydroxyprogesterone caproate (17-OHP-C) 250 mg/week). The list of specific treatment recommendations and the attribution, which guideline used them, is shown in Table 2.

**Table 2:**
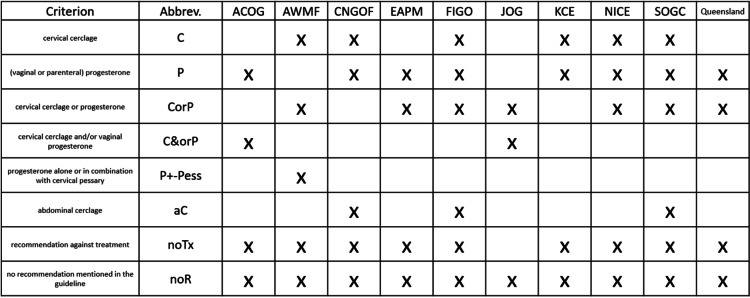
Treatment recommendations represented in individual guidelines

**Fig. 1:**
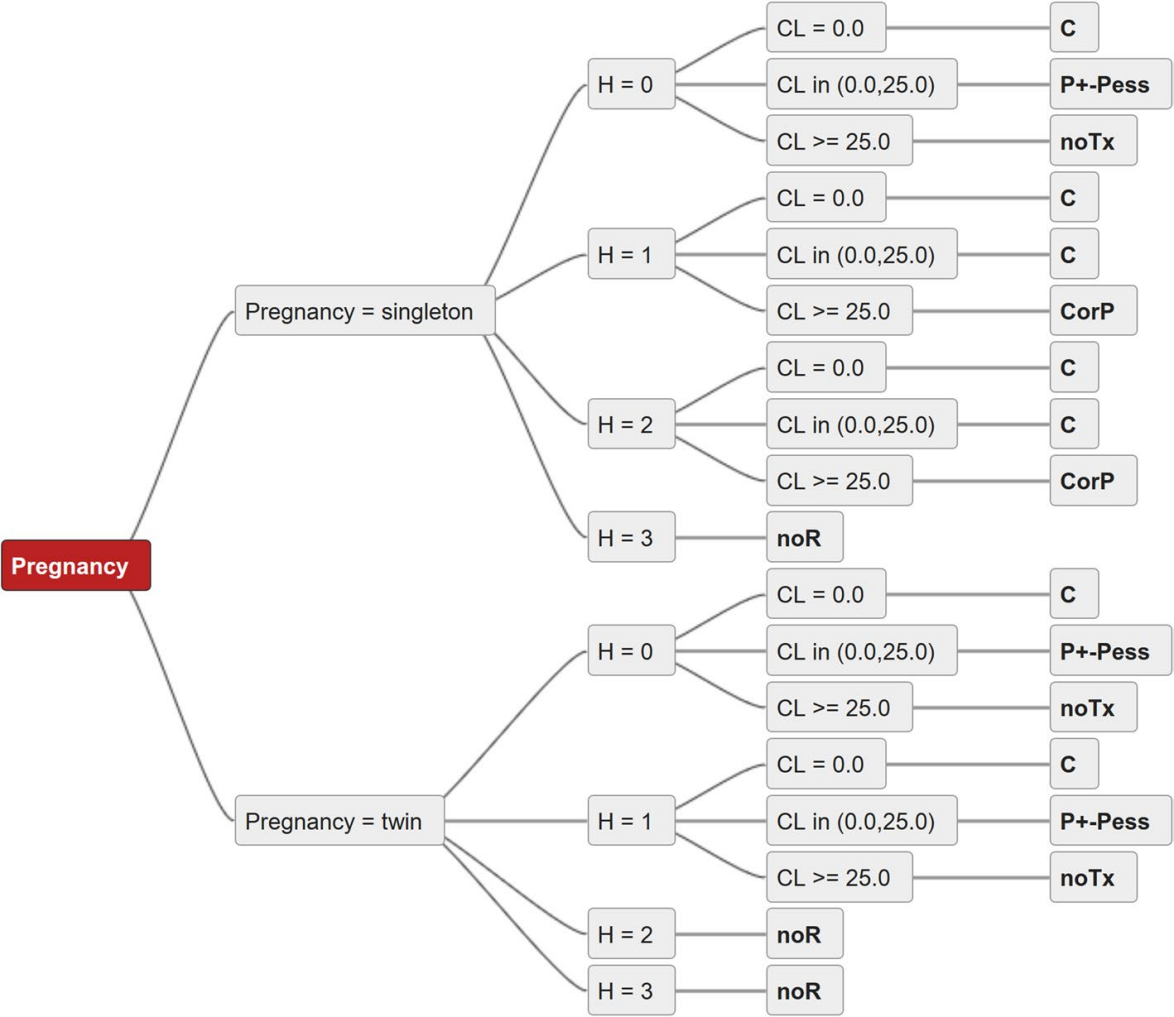
sample decision tree (based on the AWMF guideline), pregnancy = singleton vs. twin, H - history, CL – cervical length in mm, CL = 0.0 – cervical opening 1-4 cm, C – cerclage, P – progesterone, Pess – pessary, noTx – no treatment, noR – no recommendation. CorP - cerclage or progesterone, P + -Pess progesterone with or without cervical pessary

Discrepancies in the interpretation of the specific recommendations in the ten guidelines between the specialists, which could be interpreted in different ways, were discussed among KP and JK and a consensus was found. The resulting decision trees were compared using the objective consensus methodology [26, 31]. During this process each possible combination of decision-making criteria is investigated for each individual decision tree (guideline), the collected decision criteria for these combinations are then evaluated to establish the most common answer or to visualize discrepancies. Level of consensus was calculated as the percentage of guidelines recommending the most common specific treatment (or combination) divided by the number of guidelines. A semi-automatic software tool developed in Java was used to perform the comparisons [26, 31]. A sample decision tree is shown in Fig. 1.

## Results

**Fig. 2:**
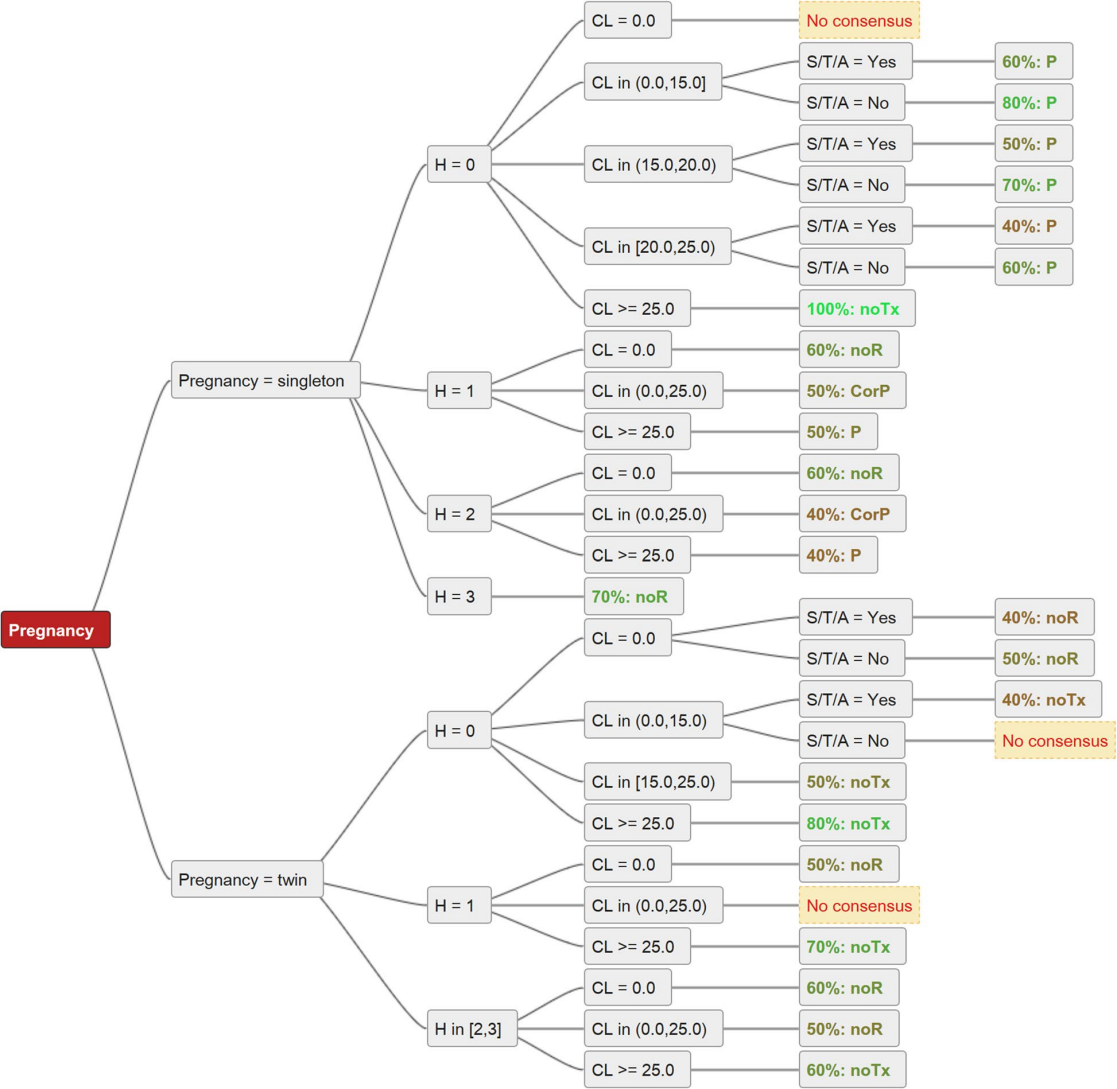
Majority recommendations for combinations of pregnancy type (singleton vs. twin), history, cervical length and history of cervical surgery/trauma, no consensus – no single most common recommendation identified

The only full consensus was shown for singleton pregnancies with no history of preterm birth and long cervix (SP, H0, CL ≥ 25 mm), where all ten guidelines agree that no prophylactic therapy should be applied (Fig. 2).

In a case of a singleton pregnancy with H0 and short cervix (CL < 25 mm), most guidelines recommend progesterone therapy. The recommendations only vary by the cutoff of the cervical length (cutoff < 25, < 20 and < 15 mm, respectively) (Fig. 2).

**Fig. 3:**
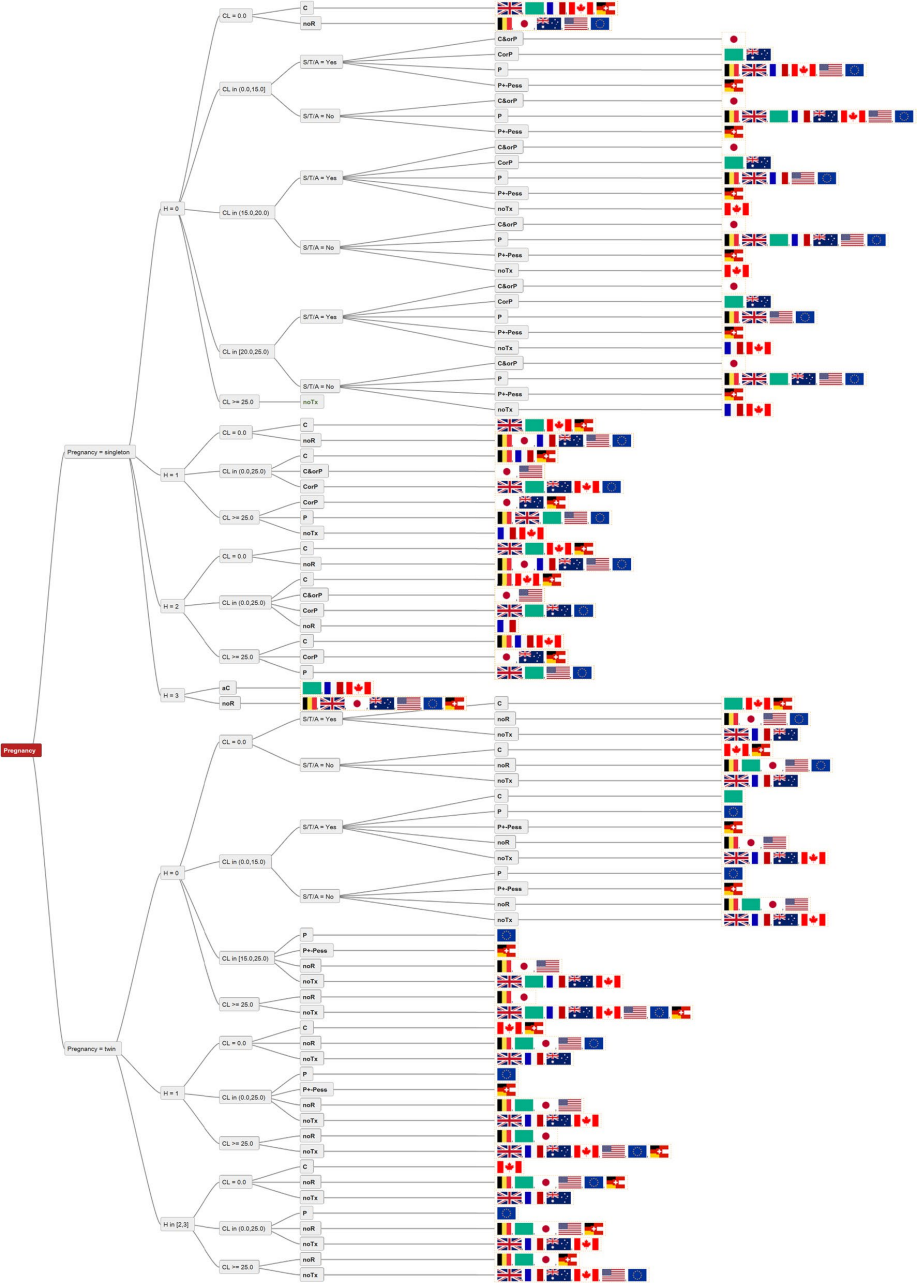
Depicts all guideline recommendations in a decision tree. Some guideline groups cover only singleton pregnancies, while others also cover twin pregnancies. All, except for the NICE guideline, exclude triplets (or more) from their recommendations. Specific treatment recommendations based on combinations of type of pregnancy, history, and cervical length. Pregnancy = singleton vs. twin, H – history, CL – cervical length in mm, CL = 0.0 – cervical opening 1-4 cm, S/T - history of cervical surgery or trauma. C – cerclage, P – progesterone, Pess – pessary, noTx – no treatment, noR – no recommendation. CorP - cerclage or progesterone, P + -Pess progesterone with or without cervical pessary. The individual guidelines are represented by their regional flags, FIGO by a green flag

In a singleton pregnancy with H1 and CL < 25 mm, all guidelines recommend a cerclage in some form or another. Nevertheless, in this situation, no single clear consensus could be demonstrated, as the guidelines leave it as an option, alternative or conjunct to progesterone therapy (Fig. 3).

In case of cervical opening, all guidelines, mentioning this specific situation, agree on rescue cerclage (Fig. 3).

The majority of the guidelines advise against prophylactic or therapeutic treatment in twin pregnancies with or without a history of preterm birth (H0 and H1) and CL ≥ or < 25 mm. The other guidelines recommend P with or without Pess, cerclage in case of risk factors (S/T/A yes) or abstain from a recommendation.

There is consensus on the recommendation towards abdominal cerclage after failure of cervical cerclage or after cervical amputation. However, only four out of ten guidelines address this issue at all (Fig. 2).

Progesterone (P) and Cerclage (C) have been considered as treatment options in every guideline, however not always for the same conditions. Eight out of ten guidelines recommended only progesterone treatment and six out of ten guidelines saw specific indications for a cerclage as monotherapy. Seven out of ten guidelines left the decision to the physician to choose between C and P (Table 2). The option of combining a cervical pessary (Pess) with P was only mentioned in the AWMF guideline, and ACOG and the Japanese guideline were the only ones mentioning the possible combination of C and P represented as C&orP. ACOG and CNGOF used the history of cervical surgery/trauma or anatomical anomaly (S/T/A) as an indication for cerclage (Table 2).

## Discussion

Choosing the most appropriate course of action for the risk of preterm birth before or around viability is a challenge. Evidence-based guidelines aim to assist the obstetrician in decision-making in clinical practice. We investigated consensus and differences among international, national, and regional guidelines to obtain a better overview of current recommendations in the setting of increased risk for preterm birth. Most guidelines propose different therapeutic options for most situations and often a single definitive recommendation is missing, leaving the decision most often up to the clinician.

There is no general agreement, whether or not a pregnancy should be monitored by routine serial cervical measurements. While unnecessary interventions may emerge from routinely performed measurements on the one hand, early interventions may positively influence the outcome of the pregnancies on the other hand.

Overall, we face multiple options and significant differences between established guidelines. Things become even more complicated as there may be various definitions on basic terms such as cervical shortening. In fact, cut-off values for cervical shortening used by various guidelines ranged from 15 to 25 mm (Fig. 3).

### Main findings

Most guidelines recommend progesterone (P) treatment for cervical shortening, for patients without a history of preterm birth. Those patients, who suffered from a previous preterm delivery, should be offered mainly a cervical cerclage (C). This result is supported by two recent meta-analyses. Romero et al. showed that vaginal progesterone decreased the risk of preterm birth in singleton pregnancies with a shortened cervix, when compared to placebo [32]. Likewise, Berghella et al. demonstrated that a cerclage reduced preterm birth among singleton gestations with short cervical length, especially for those patients with a prior preterm birth [33].

The latest gestational week that may be suitable for the insertion of a cervical cerclage is still under debate. ACOG, AWMF, CNGOF, EAPM, KCE, SCOG, QL suggest that the cerclage should not be inserted later than 24 weeks of gestation (while NICE sees an indication for it up to 27 + 6 weeks of gestation).

Most guidelines recommend P for patients with a history of preterm birth (H1) and CL ≥ 25 mm. A recent Cochrane systematic review supported this recommendation. The authors report, that the preventive application of progesterone for women with a previous preterm birth reduced the risk of preterm birth before 34 gestational weeks, reduced perinatal mortality, reduced the incidence of low birthweight, and reduced neonatal death. In addition, neither the route of administration, nor the gestational week, in which the treatment began, affected the pregnancy outcome [34]. Preventive progesterone treatment is typically initiated around the end of the first trimester (i.e. around 12–14 weeks of gestation), or when cervical shortening is first detected. The end of application ranges from 34 to 37 weeks of gestation (KCE, QL, CNGOF, AWMF). Some guidelines do not explicitly define the end of treatment.

Interpretations: In most recommendations, the route of application of progesterone is vaginal. A recent double-blind, placebo-controlled international trial demonstrated, that intramuscular injection of 17-OHP-C did not decrease recurrent preterm birth < 35 weeks of gestation in comparison to placebo [35]. Therefore, vaginal progesterone seems to be superior to the intramuscular application, reflecting the guidelines majority recommendation. Beside this, the European Medicines Agency (EMA) did not approve 17-OHP-C for clinical use, whereas the US Food and Drug Administration (FDA) did not approve micronized progesterone, explaining the different recommendations in the guidelines.

Cervical opening is a critical event, especially before viability. As soon as the amniotic membranes are exposed to the vaginal flora, the process of destruction begins with the risk of premature rupture of membranes. By the surgical closure of the cervix, delivery may be postponed by 4–9 weeks [36–38]. All guidelines addressing this specific situation, recommend a physical examination indicated cerclage for cervical opening under the condition that the cervix is not dilated more than 4 cm, the patient has no contractions and there is no sign of infection.

Although a twin pregnancy is a risk factor for preterm birth, most guidelines do not advise treatment, even if the cervix shortens. Based on a systematic review of randomized trials, a cerclage cannot currently be recommended for clinical use in twin pregnancies. In cases of cervical opening before 24 weeks of gestation, an emergency cerclage may be considered and discussed with the patient [39]. A recent meta-analysis did not support the use of cervical pessary to prevent preterm birth or to improve perinatal outcomes in twin gestations with a short cervix [40]. Nevertheless, a systematic review demonstrated that the administration of vaginal progesterone to asymptomatic women with a twin gestation and a sonographic short cervix in the mid-trimester reduced the risk of preterm birth significantly between 28 and 34 gestational weeks [41].

Most guidelines do not recommend the combination of more than two of the three therapeutic options (P, Pess and C). This is supported by a Cochrane review [42].

Interestingly, the only guideline mentioning total cervical occlusion (TCO) is the AWMF guideline and the only two guidelines mentioning cervical pessary are the AWMF and FIGO guidelines. These two options are not shown in our decision tree. Multiple randomized clinical trials (RCTs) have been published in the last years on the use of cervical pessary during pregnancy. However, the results remain contradictory. A Cochrane meta-analysis did not show, that the use of a cervical pessary in early pregnancy prevented preterm delivery [40]. Despite this, the cervical pessary remains popular in German speaking countries. For total cervical occlusion only small case series exist without a clear benefit [43]. This might be the reason, why it is not included in other guidelines.

### Strengths and limitations

The main limitation of the present analysis is the potential misinterpretation of guideline statements by the two obstetricians involved. Furthermore, results of our analysis may be biased by the selection of guidelines. However, the authors believe that most important guidelines have been included into this study, while adding or removing guidelines from the list would not affect the conclusion.

We did not challenge the methodological quality of the analyzed guidelines and all guidelines were equally weighted in our analysis. The publication dates of the guidelines were also not considered in the analysis (beyond as an initial selection criterion).

Clustering patients into the four groups H0-H3 is arbitrary. A more detailed risk-based classification (e.g. based on the week of preterm delivery or on comorbidities of the patient) has the potential to increase comparability between guidelines.

To our knowledge, this is the first analysis of its kind in the management of pre-term birth.

## Conclusion

A shortened cervix and or a history of preterm birth under 34 weeks of gestation is relevant in the decision-making process. In case of cervical shortening without a history of preterm birth, guidelines generally recommend the application of progesterone until 34–36 weeks of gestation. In case of cervical shortening in combination with a history of preterm birth a cervical cerclage is the most commonly recommended therapy. In case of cervical opening, a physical examination indicated cerclage should be considered in the absent of contractions or signs of tripe I. In case of unsuccessful vaginal cervical cerclage or after earlier cervical amputation, an abdominal cerclage is a therapeutic option. In general, the combination of two of the three therapeutic options (progesterone, cervical pessary and cervical cerclage) or the use of a cervical pessary is not recommended. In the setting of twin pregnancies, most guidelines recommend abstention from active treatment.

## Data Availability

The datasets used and/or analysed during the current study available from the corresponding author on reasonable request.
