# Correction: Progesterone, cervical cerclage or cervical pessary to prevent preterm birth: a decision-making analysis of international guidelines

**DOI:** 10.1186/s12884-022-04744-6

**Published:** 2022-05-16

**Authors:** Katharina Putora, René Hornung, Janis Kinkel, Tina Fischer, Paul Martin Putora

**Affiliations:** 1 Department of Gynecology and Obstetrics, Kantonsspital St. Gallen, St. Gallen, Switzerland; 2 Department of Radiation Oncology, Kantonsspital St. Gallen, St. Gallen, Switzerland; 3 Department of Radiation Oncology, University of Bern, Bern, Switzerland

**Correction: BMC Pregnancy Childbirth 22, 355 (2022)**

**https://doi.org/10.1186/s12884-022-04584-4**

Following publication of the original article [1], the authors identified an error in the author name of Katharina Putora, René Hornung, Janis Kinkel, Tina Fischer, and Paul Martin Putora. The given name and family name were erroneously transposed.

The incorrect author name is: Putora Katharina, Hornung René, Kinkel Janis, Fischer Tina, and Putora Paul Martin.

The correct author name is: Katharina Putora, René Hornung, Janis Kinkel, Tina Fischer, and Paul Martin Putora.

The author group has been updated above and the original article [1] has been corrected.
